# Using construction procurement strategy to achieve socioeconomic development objectives

**DOI:** 10.1016/j.heliyon.2024.e33537

**Published:** 2024-06-25

**Authors:** Samuel Laryea, Ron Watermeyer

**Affiliations:** School of Construction Economics and Management, University of the Witwatersrand, Johannesburg, South Africa

**Keywords:** Construction procurement strategy, Document analysis, Secondary procurement objectives, Social value, Socioeconomic development outcomes, South Africa, Targeting strategy

## Abstract

The procurement strategy for a construction project should provide the framework to achieve secondary procurement and socio-economic development objectives [2]. However, little attention has been focused on this in theory and practice. This paper addresses that gap by presenting a case study of the innovative targeting strategy developed and successfully implemented on a New Universities Project in South Africa to promote specified socioeconomic development objectives. Document analysis was used to examine how four socioeconomic development targets or key performance indicators, namely: local employment, skills development, local expenditure, and B-BBEE, were contractually integrated into the main works contracts. Four out of five framework contractors achieved the development targets, with low-performance damages applied in one case where the contractor failed to achieve all the development targets. The findings demonstrate how an appropriate construction procurement strategy that effectively integrates the packaging, targeting, and contracting strategies with effective systems for monitoring performance-based specifications, can play an essential role in promoting and realising socio-economic development objectives and social value through infrastructure projects.

## 1 Introduction

Construction projects generally promote two broad objectives in any geographic location [1]. The first one relates to the primary reasons for which the project is authorised for construction from a business case perspective. These are often referred to as the primary objectives. The second relates to any additional objectives that may be promoted through the project such as socioeconomic development objectives [2].

A close examination of the construction management literature shows that most published research papers are generally related to the primary objectives of projects such as cost, time, quality, delivery and performance issues. Relatively less attention has been focused on the secondary objectives aspects relating to how construction projects promote socioeconomic development issues in their geographic locations.

In the current literature, there is little empirical research on how secondary procurement objectives are specifically integrated into construction contracts, and actively promoted to achieve socioeconomic development objectives. This is the specific research gap this paper aims to address using evidence from a case study that entails innovative ways in which secondary procurement objectives were integrated into the contractual framework of an infrastructure project. The case study examined is the New Universities Project in South Africa (hereafter referred to as NUP), which was started in 2014 and remains ongoing until 2025 to provide two new universities, one in each of two Provinces that previously had no universities. The innovative socioeconomic development goals formulated as part of the procurement strategy for this public infrastructure project provided an opportunity to examine this case and contribute empirical evidence to the wider discussions around using public procurement for socioeconomic development outcomes.

In recent years, there has been an emergence of research publications on topics such as social procurement and social value in construction, which have emerged in response to the broader sustainability agenda. Such papers, which include Loosemore [3], Murphy and Eadie [4], Loosemoore et al. [5] and Gidigah et al. [6], focus mainly on the incorporation of social sustainability principles in construction projects. No major empirical research has been conducted to specifically examine the integration of secondary procurement or socioeconomic development objectives into infrastructure projects to determine how these were contractually integrated and the specific socioeconomic development outcomes achieved by the parties. This paper addresses this existing gap in the research literature. Furthermore, no study has yet examined this from a construction procurement strategy perspective. This is also another major gap in the literature that this paper addresses.

As this paper will demonstrate, the procurement strategy for an infrastructure project should provide the essential framework to achieve secondary or social procurement objectives. ISO 22058 [2] describes a construction procurement strategy as the packaging, contracting, pricing, targeting, and tendering strategies for any procurement. According to ISO 22058 [2], a targeting strategy helps to promote social or secondary procurement objectives, which are those additional to the immediate or primary procurement objectives. This may include the development of local enterprises, employment creation, local economic development, skills development, environmental standards, sustainability, and other areas [1].

Therefore, the research aim was to examine and analyse the secondary procurement objectives and targeting strategy for the NUP, and how this was successfully implemented to promote the intended socioeconomic development goals.

There were three specific objectives outlined as follows.

• Examine the secondary procurement policy and targeting strategy formulated to promote secondary procurement objectives;
• Examine the contractual framework used to integrate the secondary procurement objectives or socioeconomic development targets into the main works contracts as key performance indicators (KPIs) for contractors; and
• Evaluate the achieved outcomes of the socioeconomic development targets.

The findings provide theoretical evidence and insights on the role of collaborative contracts and contractual instruments in achieving social value in construction and practical insights on how socioeconomic development targets can be integrated into contracts for infrastructure projects.

## 2 Literature review

A review of research literature on targeted or preferential procurement practices to promote secondary procurement objectives in infrastructure projects, and concepts such as the social value of construction and socially responsible procurement (SRP), is presented.

ISO 10845-1 [1] defines a targeted procurement procedure as “the process used to create a demand for the services or goods of, or to secure the participation of, targeted enterprises and targeted labour in contracts in response to the objectives of a secondary procurement policy”. It then defines a secondary procurement policy as the aspects of a “procurement policy that promotes objectives additional to those associated with the immediate objective of the procurement itself”.

The research literature (summarised in Appendix 1) shows that targeted and preferential procurement practices have been used for decades by public construction clients in Australia, Canada, the European Union, South Africa, the UK, and the USA to promote socioeconomic development objectives. In the USA, for example, firms have for several years been legally required to include disadvantaged groups in public contracts [7]. The empirical evidence from South Africa in a paper by Watermeyer [8] and Watermeyer et al. [9] shows how the use of targeted procurement has played an essential role in promoting objectives such as poverty alleviation, job creation, and the empowerment of previously disadvantaged sectors of society since 1996.

In some parts of the literature such as Murphy and Eadie [4], Loosemore [3], and Gidigah et al. [6], alternative terminologies such as the social value of construction, social construction, and socially responsible procurement (SRP) have been used to characterise utilizing government expenditure on construction procurement to generate social value from construction activities. For example, the study by Murphy and Eadie [4] shows a practice in Northern Ireland construction procurement where SRP obligations and social policy statements have been integrated into the tendering process of infrastructure projects. Clients would require contractors to specify their plan to provide employment opportunities to people from target groups. Recent literature continues to demonstrate how legislation and contractual obligations are increasingly being used as a framework to achieve social value and socioeconomic development goals in the implementation of infrastructure projects. Based on 16 interviews with senior managers, the study by Loosemoore et al. [5] showed that ‘coercive isomorphism’ was one of the most effective drivers of social procurement adoption in Australian construction projects. This finding shows the importance of structuring any socioeconomic development requirements in infrastructure projects in contractually enforceable formats to make them effective and more likely to be achieved by the responsible parties.

In recent years, social procurement has been driven by the global sustainable development agenda as well as legislation that encourages a focus beyond the primary objectives (e.g. the UK's Social Value Act, 2012, European Union public procurement directives, 2014). In the South African context, two pieces of national legislation provide the framework for targeted and preferential procurement designed to promote redress and socioeconomic development goals in general economic activities including infrastructure procurement and delivery. These are the Broad-Based Black Economic Empowerment (hereafter referred to as B-BBEE) Act (Act 53 of 2003); and the Preferential Procurement Policy Framework Act (hereafter referred to as PPPFA) (Act No 5 of 2000) together with their subsequent amendments and regulations. According to Reddy and Rampersad [10], the primary goal of this legislation is to increase the opportunities available to historically disadvantaged people to participate in the management, ownership, and control of the country's economy. Ultimately, this is expected to reduce income inequalities and transform South Africa's economy. The Department of Trade and Industry in South Africa defines preferential procurement as the procurement of goods and services from businesses with favourable B-BBEE scorecards, small and micro businesses, and previously disadvantaged individuals or businesses (as a percentage of total procurement). However, in examining the application of preferential procurement as a tool to promote redress, Reddy [11] also highlights the need to align this socioeconomic development imperative with the constitutional right to equality.

The EU procurement regulations and UK law mentioned share some common features with the highlighted South African legislation and regulations when it comes to using procurement to promote social value and socioeconomic development goals. While South Africa's history and national development imperatives strongly inform procurement regulations, some procurement approaches are also informed by international regulations, standards, and practices. Therefore, due to similarities in the way different countries seek to achieve social value through procurement, the case study in this paper presents an example from which others can learn from the successful and innovative infrastructure procurement approach.

Following a review of procurement policy and practices in three countries and the European Union, McCrudden [12] explains that whereas “The use of public procurement to achieve social outcomes is widespread, detailed information about how it operates is often sketchy and difficult to find”. To further confirm the continuing existence of this gap in the research literature, a survey of 50 Northern Ireland construction organisations which was conducted by Murphy and Eadie [4] to ascertain perceptions of socially responsible procurement practices, similarly concluded with a suggestion on the need for more comprehensive evaluation and understanding of the tangible outcomes of socially responsible procurement. This further demonstrates the need and relevance of the research reported in this paper.

ISO 10845 [1] specifies different performance-based specifications to help formulate an appropriate contract participation goal (CPG) for, for example, targeted local enterprises, resources, and labour. Such specifications can form the basis for determining the extent to which a supplier has achieved the CPG. NUPMT [13] presents an evidence-based example of how CPGs can be achieved in construction contracts.

Secondary procurement objectives or social value can also be achieved through four types of targeted procurement procedures that can be adopted when formulating a construction procurement strategy [14]. This may be done in the form of (i) evaluation points which gives weightings to both socioeconomic and commercial factors; (ii) incentives for measurements and achievements of strategic socioeconomic development goals/targets referred to as the key performance indicators [15]; (iii) mandatory subcontracting where contractors are contractually required to engage targeted enterprises to perform specified portions of the works; and (iv) contractual obligations that require contractors to perform specified socioeconomic development targets.

The literature review demonstrates widespread acknowledgment of the potential to use procurement as a tool to promote socioeconomic development objectives, as well as the increasing incorporation of socioeconomic requirements into infrastructure projects (see Appendix 1). This is growing in importance with the increasing focus on themes such as socially responsible procurement, social value in construction, and social construction (see, for example, Loosemoore et al. [5]; Murphy and Eadie [4]. However, little empirical research has been conducted on how this is actually integrated into construction works contracts and the associated socioeconomic development outcomes. This paper addresses that gap using the empirical evidence from the innovative targeting strategy in the NUP.

## 3 Research design and methods

The research aim was to examine and analyse the secondary procurement policy and targeting strategy for the NUP and how this was successfully implemented to promote socioeconomic development goals. A case study was considered to be the appropriate research strategy that provides a comprehensive means to achieve this aim using different sources of empirical data from the specified project (Saunders et al., 2019).

### 3.1 Case study description

A brief description of the NUP is necessary to provide context. The project comprised the development of two new universities namely the Sol Plaatje University (hereafter referred to as SPU) and the University of Mpumalanga (hereafter referred to as UMP). The SPU is located in Kimberley, which is the capital of South Africa's Nothern Cape Province and the UMP is located in Nelspruit/Mbombela, which is the capital of South Africa's Mpumalanga Province. The political and geographic contexts of this public project made it imperative for the Client, which was the Department of Higher Education and Training (DHET) and the New Universities Project Management Team (hereafter referred to as NUPMT) to take the socioeconomic development aspects of the project into account to a significant degree in the project planning and implementation.

This imperative was reflected in the Outline Construction Delivery Strategy for the project. The focus of this paper relates to the secondary procurement policy and socioeconomic development outcomes of Phase I of the project. While the infrastructure development programme remains ongoing until 2025 to accommodate the planned increases in student numbers at the two universities, the scope of this paper is limited to the first phase which was scheduled for the 2016 and 2017 academic years, and for which the NUPMT was responsible. This phase of construction, which established the core campuses at each university, comprised about 16 new multi-storey buildings estimated to cost approximately R1.5 billion.

### 3.2 Data collection method

The primary research method was document analysis. The main reason for the choice of document analysis as the appropriate data collection method was the nature of the research objectives and the consideration that document analysis is an intense method that will generate findings with a high degree of internal and ecological validity in comparison to alternative methods such as interview and questionnaire, which would have yielded data from the subjective qualitative information provided by respondents as opposed to accurate and reliable project information available from official documents that were accessible. According to Bowen [16], “Document analysis is a systematic procedure for reviewing or evaluating the information in documents”. As a theoretical framework for applying the document analysis method, Morgan [17] discussed four factors to consider when selecting documents for a document analysis study namely: authenticity, credibility, representativeness, and meaning. In the current study, authenticity, which refers to the extent to which a document is genuine, was achieved by obtaining the relevant documents directly from the NUPMT which was the implementing agent for the project (see Table 1). Credibility, which refers to the extent to which documents are free from errors, was ensured by using the final version of documents that were included in the official close-out report of the project. representativeness, which refers to how typical a document is, was achieved by analysing the actual project documents. Meaning, which refers to the significance of a document's content, was achieved by examining all documents about the project to gain both specific and contextual information for the proper interpretation of the information.

**Table 1:** Documents examined for the data collection.

No.	Documents examined	No of pages	Key information collected
01	Outline Construction Delivery Strategy	2	Strategic goals to be promoted with the procurement
02	NUPMT Close out report Chapter 9 on “Procurement Strategy” with its eight separate supporting documents	354	Adopted packaging, contracting, pricing, targeting, selection strategies for the project
03	NUPMT Specification for local participation in engineering and construction contracts	3	Requirements for expenditure on the employment of local sub-contractors and suppliers as well as labour
04	NUPMT Specification for B-BBEE spend in engineering and construction contracts	4	Requirements for B-BBEE spend on procurement
05	NUPMT Specification for direct employment generated in engineering and construction contracts	3	Requirements for local employment
06	NUPMT Specification for developing skills that result in nationally accredited outcomes through infrastructure contracts	16	Requirements for development of skills and qualifications
07	NUPMT Close out report Chapter 11 on “Client focus on development” with its nine separate supporting documents	96	Socio-economic development requirements and targets for contractors, and achieved outcomes of the development targets by contractors
08	List of key Stakeholder meetings and presentations	15	Stakeholder engagement mechanisms and consultations
09	Construction moves up a gear – A briefing note on the construction programme for the Sol Plaatje University (SPU)	15	Progress on SPU construction works and socio-economic development targets
10	Construction moves up a gear – A briefing note on the construction programme for the University of Mpumalanga – UMP	17	Progress on UMP construction works and socio-economic development targets
11	On Site – construction programme update – SPU	17	SPU construction works and socio-economic development targets

### 3.3 Sampling method

Morgan [17] explains that while the appropriate number of documents may not be possible to determine in advance, researchers must nevertheless decide on an appropriate sampling technique for the selection of documents that will help achieve the research objectives. In the course of pursuing the research, a researcher is likely to encounter a broad range of documents and all the ones that are directly relevant to the research aim and objectives would typically need to be considered for examination and analysis. A key sampling method suggested by Flick [18] for document analysis is purposive sampling. Document analysis requires the proper examination and interpretation of evidence to elicit meaning, gain understanding, and develop empirical knowledge from the information collected from documents (see Table 1) [16]. Thematic analysis may then be used as a method of data analysis where the data is qualitative [17].

### 3.4 Research data

From the methodological guidelines above, it was determined the main data required to address the research objectives needed to be authentic, credible, representative, and meaningful project information. Therefore, the necessary project information and documents required to address these imperatives were considered to be the actual documentation approved by the client and implementing agent organisation, that is, the NUPMT, and used for procurement and implementation of the project. The list of documents collected and examined to satisfy these imperatives and address the research objectives is summarised in Table 1. The specific project information extracted from each document for this paper is also summarised in Table 1.

The data source was the comprehensive close-out report for Phase I [13] comprising of 313-pages (15 chapters) together with 124 separate supporting documents. The full close-out report, and its attachments, which contain the evidence used in this paper, are publicly available at the following link on the university's website: https://www.wits.ac.za/ipdm/evidence-based-publications/close-out-report/with the NUPMT's approval for this infrastructure procurement and delivery management (IPDM) body of knowledge to be used internationally for educational and research purposes. Thus, the data used for this study was credible existing documentary data sources rather than primary data taken directly from human subjects or participants. Use of the publicly available data on the website above is duly and ethically acknowledged and has also been acknowledged in the data availability statement.

### 3.5 Analytical themes

In terms of the development of the analytical themes, Table 2 shows the five analytical themes that were generated from ISO 10845 [1] (the international standard for construction procurement) and ISO 22058 [2] (the international standard for construction procurement strategy and tactics). These international standards provide the five components of a construction procurement strategy used as analytical themes to identify and examine the adopted procurement strategy for the NUP. The four analytical themes in Table 3, Table 4, Table 5, Table 6, Table 7, which helped to probe deeply into the secondary procurement objectives of the NUP and the procurement strategies of the NUPMT, were generated from the four KPIs stated in the construction procurement strategy (referenced as Document 01 in Table 1) as the specific socioeconomic development objectives on the NUP.

**Table 2:** Adopted procurement strategy for the delivery of new buildings and associated site works.

Aspect of strategy	Adopted strategy
**Packaging strategy**	**Brief description of the package** • **First phase facilities under the NUPMT for third intake of student** **UMP:** 6153 m^2^ student residence; 2123 m^2^ lecture halls and 7536 m^2^ building comprising academic and administrative spaces. **SPU:** 12 474 m^2^ 342 bed student residence, 13 532 m^2^ multipurpose building including a dining hall and kitchen, ground floor retail space and student residence; 9624 m2 academic building including raked lecture theatres and site infrastructure serving the three buildings • **Second phase buildings under the newly appointed SPU and UMP staff** A range of academic and administrative facilities supporting the roll out of the academic enrolment plan (approximately R 1,3 billion per annum budget split between SPU and UMP **Framework agreement:** 3 year
**Contracting strategy**	**Contract type:** construction contract **Standard form of contract:** NEC3 Engineering and Construction Contract (Option C)
	**Pricing strategy:** Target contract with activity schedule
**Design and interface management strategy**	**Design strategy**: design by client **Interface management strategy**: general contractor
**Targeting strategy**	• Granting of tender-evaluation points in the form of a preference for level contribution to Broad-Based Black Economic Empowerment • Evaluation points in expressions for level of contribution to Broad-Based Black Economic Empowerment • Creation of contractual obligations (see Table 2)
**Selection method**	Restricted competitive negotiations

**Table 3:** Socio-economic development KPIs and Standard specifications.

KPI	KPI definition	NUPMT Standard specifications
**Contract local participation goal (CLCG)**	The percentage of the Defined Cost excluding amounts for specialist subcontractors included in the amount due following Completion of the whole of the works	Specification for local participation in engineering and construction contracts
**Broad-based black economic empowerment spend goal (B-BBEE SG)**	The Contractor's total B-BBEE procurement spend to Provide the Works, expressed as a percentage of the Contractor's total procurement spend	Specification for B-BBEE spend in engineering and construction contracts
**Contract local direct employment goal (CLDEG)**	The percentage of the total number of equivalent person days worked by people employed by the Contractor or a Subcontractor within the Working Area who are local people	Specification for direct employment generated in engineering and construction contracts
**Contract skills development goal (CSDG)**	The number of hours of skills development opportunities that a contractor contracts to provide in relation to work directly related to the contract or order	Specification for developing skills that result in nationally accredited outcomes through infrastructure contracts

**Table 4:** Construction development targets (KPIs).

Key Performance Indicators	SPU Targets	UMP Targets
1. Expenditure on the employment of local sub-contractors and suppliers as well as labour	R179 million	R200 million
2. Direct employment of local labour in terms of the number of person days worked by local people	Employment of 266 persons on average for the full contract period	Employment of 327 persons on average for the full contract period
3. Broad-based Black Economic Empowerment spend aligned with the scorecard for preferential procurement	R245 million	R186 million
4. Skills development towards nationally accredited outcomes		
**Method 1**: structured work experience learning opportunities towards a part or a full occupational qualification	21 220 Hours (2625 Days)	30 796 Hours (3422 Days)
**Method 2**: structured work experience learning opportunities for apprentices or other artisan learners	29 130 Hours (3641 Days)	47 972 Hours (5325 Days)
**Method 3**: work integrated learning opportunities for University of Technology or Comprehensive University national diploma students	25 707 Hours (3213 Days)	31 944 Hours (3549 Days)
**Method 4**: structured work experience opportunities for candidates towards registration in a professional category of registration	13 443 Hours (1680 Days)	12 834 Hours (1426 Days)

**Table 5:** Skills development.

	SPU		UMP		Examples of Qualifications
	Days	Learners	Days	Learners	
**Method 1**	8774	176	10 194	99	Scaffolding Inspector & Erector; Working at Heights; Shot fixing; Safety, Health and Environment; Banksman; First Aider; Crane Operator; Dumper Operator; and Telehandler.
**Method 2**	5585	57	7473	160	Plumber; Carpenter; Plasterer; Welder; Bricklayer; Power Floating Supervisor; Tiler; and Scaffolding/Formwork.
**Method 3**	3329	16	2636	18	National Diploma: Civil Engineering; and National Diploma: Building Science.
**Method 4**	2165	5	1381	14	Quantity Surveyor; Engineer; Construction Manager.
**TOTAL**	**19 853**	**254**	**21 684**	**291**

**Table 6:** Local expenditure.

	Total Actual Spend to Date	% Target of Local Expenditure	Actual Local Expenditure Spend	Actual %
**SPU**	R 502 312 001.95	36 %	R 188 254 116.65	38 %
**UMP**	R 237 820 000.00	44 %	R 174 130 000.00	73 %

**Table 7:** Broad-based black economic empowerment.

	Total Actual Procurement Spend	B-BBEE Target as a % of Procurement Spend	Calculated B-BBEE Procurement Spend	Actual %
**SPU**	R 423 061 711.32	60 %	R 327 919 489.66	78 %
**UMP**	R 218 910 000.00	60 %	R 195 830 000.00	89 %

### 3.6 Data analysis method

The qualitative text from documents in Table 1 was collated and examined using thematic analysis. According to Braun and Clark [19] and Braun et al. [20], thematic analysis, which involves searching across a data set to find repeated patterns of meaning, provides a means to identify, analyse and interpret patterns. The three objectives of this paper were to examine the secondary procurement policy of the project as set out by the client, the integration of socioeconomic development targets into the main works contracts, and the achieved socioeconomic development outcomes. Therefore, the data relating to these themes were collated to identify, analyse and interpret the emerging patterns. The approach was mostly semantic where interpretation was based on the explicit content of the project information summarised in Table 1. This approach is explained in Braun et al. [20].

## 4 Data analysis and findings

This section presents the data relating to the research objectives. Firstly, the data relating to the primary and secondary procurement objectives. Secondly, the data relating to specified socioeconomic development objectives, and how these were integrated into the main works contracts. Thirdly, the data relating to the extent to which the development targets were achieved by contractors. Table 1 summarises the documents that were reviewed to address the research aim and specific objectives.

### 4.1 Secondary procurement policy formulated to promote secondary objectives

The procurement objectives for the project (primary and secondary) were stated explicitly in the Outline Construction Delivery Strategy (document 01 in Table 1). This document, which was discussed with stakeholders in both UMP and SPU, highlighted the need for a construction contracting strategy that will promote four strategic goals at a high level. The first strategic goal was to ensure the construction of competent buildings and infrastructure that met the cost, time, and quality specifications. The second strategic goal was to promote empowerment, which is a secondary procurement objective. The third strategic goal was to promote local economic development through local content, local employment opportunities, and skills development. The fourth strategic goal was to ensure that the delivery strategy was supported by stakeholders. Thus, two of the four strategic goals that were specified by the client were linked to social value and the promotion of secondary procurement objectives.

The NUPMT specifically outlined six primary objectives and three secondary objectives that the project teams needed to achieve within the strategic framework [13].

The primary objectives, as stated in document 01 in Table 1 were about cost (two specific objectives relating to delivery within the control budget, and linking expenditure to the fiscal cycle and cashflow of the client); time (one specific objective which was a requirement for contractors to ensure completion of the works before the start of the academic year); and quality (three specific objectives which were about straightforward maintenance of the facilities, alignment with future best practice, and minimal maintenance costs). The secondary objectives were to promote B-BBEE, local participation and employment, and skills development through academic, vocational, and professional qualifications. The overall procurement strategy adopted for the delivery of new buildings and associated site works in the format recommended by and described in ISO 22058 [2] is set out in Table 2. The strategy relating to the primary objectives was informed by the members of the NUPMT experience in their previous delivery of a capital programme between 2008 and 2013 as described by Laryea and Watermeyer [21]. As shown in Table 2, framework agreements were an integral part of the procurement strategy which helped to achieve the secondary procurement objectives. The framework agreements between the client and contractors in both cases enabled continuity in delivery to take place once the NUPMT handed over delivery management responsibilities to the newly appointed UMP and SPU staff.

### 4.2 Integrating the secondary procurement objectives into the contracting strategy and works contracts

This section discusses the way that the secondary procurement objectives were integrated into the packaging and contracting strategy for the project. According to ISO 22058 [2], a contracting strategy refers to selecting an appropriate form of contract including the pricing strategy. Details of the contracting strategy for the project are outlined in Table 2.

The 1996 Constitution of South Africa and ISO 10845 [1] require the procurement of public infrastructure projects to be based on “fair, equitable, transparent, competitive, and cost-effective” systems. The Constitution permits a departure from these requirements in the implementation of a procurement policy providing for “categories of preference and the protection or advancement of persons, or categories of persons, disadvantaged by unfair discrimination” provided that it is done so in terms of national legislation. The PPPFA made provision for a range of goals that an organ of state could pursue through a price preference points system. However, the Preferential Procurement Regulations issued in terms of the PPPFA confined the preference points to levels of contribution to B-BBEE assessed in terms of a scorecard. Accordingly, the pursuit of any secondary objectives other than B-BBEE had to be “fair, equitable, transparent, competitive, and cost-effective” as it fell outside what was permitted in terms of national legislation.

The NUPMT recognised that framework agreements given the nature and scale of the construction works would in the main be entered into with national contractors, particularly in the case of SPU. The NUPMT furthermore was aware that the politicians saw the universities as provincial projects as the intent behind the project was to provide a university in the provinces that had no university. Preference points, although contributing to national priorities were not likely to contribute to provincial priorities or satisfy provincial expectations [13].

Recognising the importance of local participation, the NUPMT formulated four specific socioeconomic development goals/key performance indicators (KPIs) to be promoted through the main works contracts. The NUPMT developed a standard specification that enabled each of the four KPIs to be clearly understood, measurable, and capable of being implemented through works contracts (see Table 3).

The socioeconomic development KPIs in Table 3 were integrated into the NEC3 Engineering and Construction Contracts (Option C), with required contractual targets. The NEC3 ECC contains Option X17 optional clause (low-performance damages) which can be used by clients wishing to apply an incentive/penalty mechanism in the contract. The Option X17 clause with minor modifications was included in the construction works contracts as a penalty mechanism if a contractor failed to achieve the development targets.

### 4.3 Achieved outcomes of the socioeconomic development KPIs

The KPIs for both SPU and UMP were set between the project managers and the appointed contractors. This was done after the refinement of the construction costs. The KPIs are described in Table 3. Table 4 shows the agreed KPIs/development targets that were set and agreed upon by the project managers and contractors.

The remainder of this section of the paper will focus on the outcomes achieved in respect of the four development targets namely: Local employment, Skills development, Local expenditure, and B-BBEE. The data in NUPMT [13] shows that contractors mostly exceeded the socioeconomic development targets. Four out of five framework contractors achieved the development targets, with low-performance damages applied in one case where the contractor failed to achieve all the development targets.

#### 4.3.1 KP1 1: local employment

Regarding the specification for direct employment (see Table 2) and the construction development targets (see Table 4), an examination of the evidence in NUPMT [13] shows that the level of the opportunities arising from the infrastructure project for targeted groups was dependent on the progress and stages of the construction programme. For example, during the initial stages of the project where the contractors are mainly performing site preparation, earthworks, and substructure activities, there is relatively limited opportunity for local subcontractors and suppliers to become involved in the works at this stage. The scale of opportunities for subcontractors and suppliers increases as the construction programme progresses towards activities requiring their participation and supplies. The detailed timelines for the delivery of Phase I of the NUP at both SPU and UMP can be found in a paper by Laryea and Watermeyer [22] which examined the project management approach used by the NUPMT in dealing with the complexity and uncertainties associated with the project.

As shown in Table 4, the direct employment goal (defined in Table 3) was for appointed contractors to employ between 30 % and 95 % of their workforce from the local community with sub-targets for youth and women. The evidence in NUPMT [13] shows 194 people were employed on the NUP construction sites at the start of the construction programme in October 2014 (see timelines in Laryea and Watermeyer [22]). This increased significantly to 1353 by the end of March 2016 when the total expenditure had reached approximately R1.6 million. The evidence from the NUP shows that 876 local people were employed (representing approximately 65 % of total employment). Averagely, 1031 people were employed each month throughout the construction period and 654 (63 %) of them were local. There were only seven women involved at the start of the project in October 2014. However, this increased to 186 by March 2016, representing approximately 10 % of workers employed on site. The employment specification also emphasised the importance of providing opportunities to young people. The evidence examined showed that approximately 75 % of all workers on-site were young people. When compared to the development targets in Table 4, the achieved outcomes show that the targets were significantly exceeded.

#### 4.3.2 KPI 2: skills development

The NUPMT specified the requirement for contractors to provide opportunities for the development of skills that result in nationally accredited outcomes (see Table 2). This was weaved into the construction development targets (see Table 4) and works contracts. The skills development goal was essentially for main contractors to provide practical training opportunities amounting to 250 h per million and expenditure [13]. It should be noted that the Construction Industry Development Board (CIDB) in the year 2000 published a “Standard for Developing Skills through Infrastructure Contracts” which is a “best practice Standard for developing skills through infrastructure contracts. The standard establishes a minimum contract skills development goal which is to be achieved in the performance of a contract concerning the provision of different types of workplace opportunities linked to work associated with a contract which culminate in or lead to” occupational, trade, academic or professional qualifications.

The evidence in NUPMT [13] (see Table 5) shows that the construction programme at SPU and UMP provided various opportunities for 545 people to gain structured workplace learning experience towards the attainment of a nationally recognised qualification. This could also be any workplace learning opportunity that supported people to partially or fully gain an occupational, artisanal, or professional qualification. The framework shown in Table 4 provided contractors with four methods or options through which they could provide local people with structured workplace learning opportunities for the attainment of occupational qualifications, trade qualifications, diplomas, and professional registration (see Table 4).

The innovative structured workplace learning requirement imposed on contractors was designed by the NUPMT as a direct response to the difficulties faced by many learners in South Africa when it comes to finding appropriate workplaces where they can gain exposure and complete the practical experience component of their qualifications [13]. The evidence in Table 5 shows that between October 2014 and March 2016, 545 learners were provided with 41 537 days of workplace learning experience, which significantly exceeds the target set out in Table 4.

#### 4.3.3 KPI 3: local expenditure

Regarding local expenditure (see Table 2) and the construction development targets or KPIs (see Table 4), the NUPMT specified a target of between 30 % and 50 % in Kimberley, and in Nelspruit as high as 95 %. The evidence in NUPMT [13] shows that the two framework Contractors at the UMP successfully exceeded their target for local expenditure by March 2015. The two contractors at UMP made a joint commitment to achieve 44 % of construction spend on local expenditure. By April 2016, 73 % of the total amount spent by the contractors on local labour, subcontractors, and suppliers was R174 million as shown in Table 6.

In comparison to UMP, the progress of the three framework Contractors at SPU was more gradual. The SPU contractors were only able to achieve the targeted level of local expenditure of 36 % by September 2015. By the end of Phase I of the NUP in March 2016, the contractors had cumulatively achieved a local expenditure of R188 million, which represented 38 % of the total construction spend. The data in Table 6 shows that between October 2014 and March 2016, the construction works associated with Phase 1 of the new universities development project directly contributed R362 million into the local economies of the two cities.

#### 4.3.4 KPI 4: broad-based black economic empowerment

In relation to the specification for B-BBEE spend (see Table 2) and the construction development targets (see Table 4), the B-BBEE spend goal was a minimum of 60 % calculated by the scorecard for preferential procurement. In other words, contractors were required to spend 60 % of their procurement on B-BBEE. The research evidence in NUPMT [13] shows a B-BBEE spend of R195 million (89 %) and R327 million (78 %) by contractors at UMP and SPU respectively (see Table 7).

A comparison of the target and achieved amounts in Table 6 shows that contractors exceeded the targets at both UMP and SPU. One means through which this was achieved was that main contractors identified and collaborated with capable local subcontractors and suppliers. Some contractors also provided support in the form of mentoring and skills development to local contractors to help support their business growth.

## 5 Discussion of results

The research aim was to examine and analyse the way that the targeting strategy for the NUP was formulated and implemented to promote the specified socioeconomic development outcomes. The three specific objectives are discussed to provide a basis for conclusions and recommendations.

### 5.1 Targeting strategy for the socioeconomic development objectives

The first research objective was to examine the targeting strategy used to promote secondary procurement objectives. Table 2, Table 3 show the four specific socioeconomic development KPIs and targets in relation to local employment, skills development, local expenditure, and B-BBEE. A Specification standard was developed for each of the four KPIs. This provided an objective basis for the parties to monitor the implementation and progress of each KPI, and verify the extent to which a contractor had achieved the specified performance. This approach is explained in a construction procurement strategy paper by Watermeyer [14].

The research by Laryea and Watermeyer [21] on the innovative approach used for the procurement of a capital projects programme reported on three different targeting strategies used by the client. These were preferences for B-BBEE, contract participation goals, and student bursaries. The study by Adediran and Windapo [23] examining the impact of targeted procurement on the growth of contractors in South Africa showed tendering equity, preferences for B-BBEE, and mandatory subcontracting as the most frequently used methods of targeted procurement in South African construction projects. Therefore, in terms of existing practices in the empirical literature, the targeting strategy applied in the NUP goes significantly further, particularly in terms of the contractual implementation of the skills development goal and the setting of contractual targets for the four KPIs (see Table 4).

As the NUPMT was appointed as the implementing agent for the NUP, it should be noted that the nucleus of the team that delivered the Wits capital projects formed the NUPMT. They built upon their experience in the previous procurement to formulate the targeting strategy for the NUP (see Laryea [24] for the background to their appointment as implementing agent and details of the project management team). While there are similarities, the targeting strategy for the NUP is much broader in scope, more strategic, and better designed for verification and monitoring. The development targets in the NUP were applied differently also due to the greater complexity of the project, stakeholder expectations and requirements, and other dynamics due to the nature of the NUP.

The data on the implementation of the targeting strategy of the infrastructure procurement shows a balance between strategic and pragmatic aspects. The careful analysis of CIDB-registered Grade 8 and 9 contractors in the two geographic regions and the lowering of the grading requirement in Northern Cape Province by the NUPMT shows a strategic and pragmatic approach to managing the balance between a selection of appropriate contractors and the realistic prospects of participation of local contractors.

### 5.2 Contractual framework for the socioeconomic development targets

The second research objective was to identify the contractual mechanisms used to require or incentivise contractors to achieve the secondary procurement objectives, and how this was successfully implemented in contracts. See the findings presented in Table 3, Table 4

The client entered into framework contracts based on the NEC3 Engineering and Construction Contract (ECC). This essentially changed the dynamics between the client and the contractor. It aligned the objectives of the client and the contractor as a contractor in terms of the framework agreement was not guaranteed any quantum of work. Contractor performance including the delivery of development targets would be considered when issuing future package orders. This created a positive incentive to deliver on the KPIs. This discussion point highlights the critical need for inter-organisational incentive alignment when it comes to achieving construction project goals. In their study on the role of incentives in the alignment of inter-organizational business goals, Heindel and Weber [25] identified the importance of rewarding participants for fair behaviour and efficient completion of tasks. Therefore, from an incentive theory perspective, a key incentive to secure performance from contractors is the use of contracting arrangements such as framework agreements that provide a mechanism to reward contractors with additional work for satisfactory performance.

The client's use of the NEC3 ECC Option X17 optional clause (low-performance damages) to create a contractual mechanism to penalise contractors who failed to achieve the development targets, can be explained through the lens of the theory of coercive isomorphism. The fact that four out of five contractors achieved all of the development targets with only one failing to achieve all of the targets demonstrates that this was effective to a large extent. This discussion point can be related to the findings from the study on social procurement by Loosemore et al. [5] which used new institutional theory to investigate drivers of social procurement adoption in construction projects in Australia and identified coercive isomorphism as the primary determinant of social procurement policies in construction projects. According to Martínez-Ferrero and García-Sánchez [26], coercive isomorphism refers to external forces imposed to drive compliance with the rules and norms. The contractual framework imposed by the client on contractors to drive the achievement of the development targets can be characterised as an example of coercive isomorphism. Hence, similar to the findings of Loosemore et al. [5], coercive isomorphism was a key driver of the organisational behaviour of contractors regarding the extent of achievement of the socioeconomic development targets.

Another point from the literature regarding the use of low-performance damages in construction contracts needs to be made. Broome [27] explains that the low-performance damages clause should be used for a contract where the works and works information are described using a performance specification. Table 3 shows that clear specification standards were developed for each of the socioeconomic development KPIs.

It can be argued that the outcomes achieved demonstrate that the use of the secondary option ×17 clause to stimulate contractor performance was largely effective. This is particularly true when it is considered that four out of five contractors achieved the development targets in Table 4. However, Broome [27] describes four conditions for the successful use of negative incentives, or damages, and positive incentives - bonuses – under NEC3 contracts. These are clarity of the objectives, the targets, and how performance will be measured, combining both positive (bonuses) and negative (damages) incentives, and an incentive plan that neither over – nor – under-incentivises the contractor for each objective the client seems to achieve. These four conditions were largely present in the case of the contractual framework for the NUP. Broome [27] goes on to explain that such targets should not be too many and the key is simplicity hence clients should focus on the few key objectives that are important to them rather than trying to incentivise and penalise everything.

### 5.3 Evaluation of the achieved outcomes

The third research objective was to evaluate the outcomes and extent of achievement of the secondary procurement targets. The outcomes are presented in Table 5, Table 6, Table 7. Assessment of the data shows that four contractors exceeded the targets and one contractor fell short of achieving all the targets. Thus, these targets were ultimately met on all except one ‘contract at SPU, where the client had to apply low-performance damages of over R700 000.

In evaluating the outcomes of the development targets, and discussing the extent of the achievement of development targets, it should be noted that the firm integration of the targeting strategy (socioeconomic development objectives) into the packaging and contracting strategy (see Table 2, Table 3, Table 4) as a contractual requirement was a key enabler for their achievement and securing the commitment and performance of contractors. This is an important point as contractors generally respond better to contractual rather than non-contractual mechanisms (see Hughes et al. [28] and Laryea and Hughes [29]). The research of Loosemoore et al. [5] found that coercive isomorphism was the most powerful driver of social procurement adoption when it comes to the procurement of construction projects in Australia.

The second point here is in relation to the usefulness of framework agreements when it comes to the achievement of the development objectives by contractors over time. This contrasts with the traditional method of procuring per project, which is not likely to have yielded such an outcome due to its shorter-term nature. Based on the outcomes and data in this paper, it would be argued that framework agreements, or collaborative contract approaches that enable contractors to work over a longer-term duration, provide a better opportunity to achieve socioeconomic development targets, over time, compared to traditional one-off contracts. Therefore, an appropriate construction procurement strategy, which effectively integrates the packaging, targeting, and contracting strategies, can play a significant role in promoting socioeconomic development outcomes.

As the project needed to be completed before the predetermined start of the academic year to be capable of being used for the operations of the two universities, there was a risk that the main contractors would fall back on their established and trusted supply chain partners to be able to meet the completion time. The tight time frames could have undermined the intentions to promote secondary development objectives through the creation of opportunities for local subcontractors. The process of developing new relationships with local suppliers and subcontractors took time and this should be factored into the roll-out of any future projects looking to adopt a similar model. The details of issues relating to the overall project outcomes are in an empirical paper on the procurement strategy and outcomes by Laryea [24].

## 6 Conclusions

Three main conclusions are presented based on the specific objectives and discussion of findings.

Firstly, the procurement strategy for the NUP, which was summarised in Table 2, entailed an innovative targeting strategy comprising four socioeconomic development targets or key performance indicators (KPIs) which were effectively integrated into the contracting strategy and the main works contracts.

Secondly, the integration of the KPIs stated in Table 3, Table 4 into the NEC3 Engineering and Construction Contract, and the specific use framework agreements and of the Option X17 optional clause on low-performance damages provided an effective contractual framework that secured the commitment and performance of contractors. A key theoretical contribution of this study lies in the evidence provided in support of the theory of coercive isomorphism when it comes to the use of contractual mechanisms to achieve secondary procurement objectives.

Thirdly, given the outcomes presented in Table 5, Table 6, Table 7, the findings demonstrate how the formulation and implementation of an appropriate construction procurement strategy, which effectively integrates the packaging, targeting, contracting strategies, and selection methods, and is supported by an effective specification, monitoring, and verification system, can play a significant role in promoting social value and socioeconomic development outcomes.

The research evidence demonstrates the following key conditions for success: client leadership, the buy-in of stakeholders, community liaison, procurement strategy, effective integration of packaging, contracting, targeting and selection strategy, contractually enforceable targets, and effective monitoring systems for performance-based specifications.

Two key implications of the findings are presented for academic researchers and industry actors particularly clients who are the leaders of infrastructure projects. Firstly, the case study evidence demonstrates the significant power procurement provides to promote socioeconomic development outcomes in the geographic areas of infrastructure projects if there is an appropriate procurement strategy. Procurement strategy is a prerequisite for achieving intended social value outcomes in construction. Socioeconomic development goals or social value would be difficult to achieve without an appropriate construction procurement strategy specifically formulated to achieve the intended outcomes. This point has so far been missing from the literature. The second implication of the evidence is that any intended socioeconomic development objectives should be contractually integrated into the works contracts to provide a coercive framework for contractors to achieve them. Theoretically, this study supports the existing theory of coercive isomorphism as a driver of social procurement adoption. It also extends it as a driver of social procurement enforcement among contractors rather than just adoption by infrastructure clients. For industry practitioners, this study sheds light on how social procurement can be practically implemented in infrastructure contracts. It should be noted that the contractual practices examined relate to only contractors. Future research can examine the transferability of such contractual practices to other role players in the infrastructure supply chain, including professional team members, to increase social value.

The main contribution of this study lies in examining empirical evidence on the use of targeted procurement in infrastructure projects and contracts to promote social value and secondary procurement objectives. While the study probes intensely and ecologically into one case study to generate findings with a high degree of internal and ecological validity, it should be noted that these findings reflect the socioeconomic development objectives of a particular public sector client within the geographic regions of the two new universities. These limitations should be taken into account in any transferability of the findings into other contexts.

## Data availability statement

The data used for this paper is publicly available and can be accessed at: https://www.wits.ac.za/ipdm/evidence-based-publications/close-out-report/

Additional Information.

Question.

Response.

**Data Availability**.

Sharing research data helps other researchers evaluate your findings, build on your work and to increase trust in your article. We encourage all our authors to make as much of their data publicly available as reasonably possible. Please note that your response to the following questions regarding the public data availability and the reasons for potentially not making data available will be available alongside your article upon publication.

Has data associated with your study been deposited into a publicly available repository?

Yes.

Please provide the name of the repository and the accession number here.

as follow-up to “**Data Availability**.

Sharing research data helps other researchers evaluate your findings, build on your work and to increase trust in your article. We encourage all our authors to make as much of their data publicly available as reasonably possible. Please note that your response to the following questions regarding the public data availability and the reasons for potentially not making data available will be available alongside your article upon publication.

Has data associated with your study been deposited into a publicly available repository?"

https://www.wits.ac.za/ipdm/evidence-based-publications/close-out-report/

## CRediT authorship contribution statement

**Samuel Laryea:** Writing – review & editing, Writing – original draft, Methodology, Investigation, Funding acquisition, Formal analysis, Data curation, Conceptualization. **Ron Watermeyer:** Writing – original draft, Investigation, Formal analysis.

## Declaration of competing interest

The authors declare the following financial interests/personal relationships which may be considered as potential competing interests:Samuel Laryea reports financial support was provided by Construction Industry Development Board South Africa. Samuel Laryea reports article publishing charges was provided by University of the Witwatersrand Johannesburg. If there are other authors, they declare that they have no known competing financial interests or personal relationships that could have appeared to influence the work reported in this paper.

## Figures and Tables

**Table:** 

Authors	Year	Title	Source title	Vol	Iss	Pages	Country of study	Research method/Data source
Gidigah, BK, Agyekum, K, Baiden, BK	2022	Defining social value in the public procurement process for works	Engineering, Construction and Architectural Management	29	6	–	Ghana	Interviews with infrastructure officials
Loosemore, M, Alkilani, SZ, Murphy, R	2021	The institutional drivers of social procurement implementation in Australian construction projects	International Journal of Project Management	39	7	750–761	Australia	Interviews with senior managers
Murphy, M and Eadie, R	2019	Socially responsible procurement: A service innovation for generating employment in construction	Built Environment Project and Asset Management	9	1	138–152	Northern Ireland	Qualitative interview data
Adediran, A., Windapo, A.O.	2017	The influence of government targeted procurement strategies on the growth performance of construction small and medium-sized contractors (SMCs) in South Africa	International Journal of Construction Supply Chain Management	7	3	151–164	South Africa	Questionnaire Survey
Reddy, K.	2016	The evolving role of business in contributing to social justice in South Africa in terms of legislative measures	Corporate Ownership and Control	13	2	465–472	South Africa	Literature review
Liu, X., Cui, Q.	2016	Assessing the impacts of preferential procurement on low-carbon building	Journal of Cleaner Production	112		863–871	USA	Modeling
Loosemore, M	2016	Social procurement in UK construction projects	International Journal of Project Management	34	2	133–144	UK	Interviews
Bates, T.	2015	Contested terrain: The role of preferential policies in opening government and corporate procurement markets to black-owned businesses	Du Bois Review	12	1	137	USA	Document review
Watermeyer, R.	2012	A framework for developing construction procurement strategy	Proceedings of the ICE – Management, Procurement and Law	165	4	223–237	South Africa	Experiential knowledge
Bolton, P.	2010	The regulation of preferential procurement in state-owned enterprises	Tydskrif vir die Suid-Afrikaanse Reg	2010	1	101–118	South Africa	Document analysis
Bates, T.	2009	Utilizing affirmative action in public sector procurement as a local economic development strategy	Economic Development Quarterly	23	3	180–192	USA	Spending and survey data
Bates, T.	2006	Discrimination in the Chicago-area construction industry handicaps minority-owned firms	Review of Black Political Economy	33	4	7–26	USA	Secondary data and legal cases
McCrudden, C.	2004	Using public procurement to achieve social outcomes	Natural Resources Forum	28	4	257–267	UK	Document analysis
Watermeyer, R.B.	2003	Implementing preferential procurement policies in the public sector in South Africa	Journal of the South African Institution of Civil Engineering	45	3	11–21	South Africa	Experiential knowledge
Rogerson, C.M.	2000	Road construction and small enterprise development: The experience of the N4 Maputo corridor	Development Southern Africa	17	4	535–566	South Africa	Secondary data, project documentation, interviews
Watermeyer, R	2000	The use of Targeted Procurement as an Instrument of Poverty Alleviation and Job Creation in Infrastructure Projects	Public Procurement Law Review	5		226–250	South Africa	Case studies of public works contracts
Watermeyer, R.B.	1999	Socio-economic responsibilities: The challenge facing structural engineers	Structural Engineer	77	17	22–28	South Africa	Experiential knowledge
Watermeyer, R.B., Gounden, S.M., Letchmiah, D.R. and Shezi, S.	1998	Targeted procurement: a means by which socio-economic objectives can be realized through engineering and construction works contracts	Journal of the South African Institution of Civil Engineering	40	4	15–25	South Africa	Case studies of public works contracts
Bates, T., Williams, D.	1996	Do Preferential Procurement Programs Benefit Minority Business?	American Economic Review	86	2	294–297	USA	Secondary data

Note – This Table contains the reference details for the papers hence none of them is duplicated in the references list at the end of the paper.
